# DNA methylation changes and TE activity induced in tissue cultures of barley (*Hordeum vulgare* L.)

**DOI:** 10.1186/s40709-016-0056-5

**Published:** 2016-08-08

**Authors:** Renata Orłowska, Joanna Machczyńska, Sylwia Oleszczuk, Janusz Zimny, Piotr Tomasz Bednarek

**Affiliations:** 1 Department of Plant Physiology and Biochemistry, Plant Breeding and Acclimatization Institute-National Research Institute, Radzików, 05-870 Błonie, Poland; 2 Department of Plant Biotechnology and Cytogenetics, Plant Breeding and Acclimatization Institute-National Research Institute, Radzików, 05-870 Błonie, Poland

**Keywords:** Tissue culture, Barley, RP-HPLC, SSAP, MSTD, Methylation, TEs

## Abstract

**Background:**

In vitro plant regeneration via androgenesis or somatic embryogenesis is capable of inducing (epi)mutations that may affect sexual progenies. While epimutations are associated with DNA methylation, mutations could be due to the movement of transposons. The common notion is that both processes are linked. It is being assumed that demethylation activates transposable elements (TEs). Analysis of methylation changes and their relation with TEs activation in tissue cultures requires uniquely derived donor plants (Ds), their regenerants (Rs) and respective progeny (Ps) that would allow discrimination of processes not related to changes introduced via in vitro cultures. Moreover, a set of methods (RP-HPLC, SSAP, and MSTD) is needed to study whether different TEs families are being activated during in vitro tissue culture plant regeneration and whether their activity could be linked to DNA methylation changes or alternative explanations should be considered.

**Results:**

The in vitro tissue culture plant regeneration in barley was responsible for the induction of DNA methylation in regenerants and conservation of the methylation level in the progeny as shown by the RP-HPLC approach. No difference between andro- and embryo-derived Rs and Ps was observed. The SSAP and MSTD approach revealed that Ds and Rs were more polymorphic than Ps. Moreover, Rs individuals exhibited more polymorphisms with the MSTD than SSAP approach. The differences between Ds, Rs and Ps were also evaluated via ANOVA and AMOVA.

**Conclusions:**

Stressful conditions during plant regeneration via in vitro tissue cultures affect regenerants and their sexual progeny leading to an increase in global DNA methylation of Rs and Ps compared to Ds in barley. The increased methylation level noted among regenerants remains unchanged in the Ps as indicated via RP-HPLC data. Marker-based experiments suggest that TEs are activated via in vitro tissue cultures and that, independently of the increased methylation, their activity in Rs is greater than in Ps. Thus, the increased methylation level may not correspond to the stabilization of TEs movement at least at the level of regenerants. The presence of TEs variation among Ds that were genetically and epigenetically uniform may suggest that at least some mobile elements may be active, and they may mask variation related to tissue cultures. Thus, tissue cultures may activate some TEs whereas the others remain intact, or their level of movement is changed. Finally, we suggest that sexual reproduction may be responsible for the stabilization of TEs.

## Background

There is a growing body of evidence that even morphologically identical plants regenerated via tissue culture may not be uniform at the (epi)genetic level [1], and the regenerants may differ from their donors [2, 3]. These differences can be due to changes in DNA sequence and methylation patterns. Plant genomes are usually highly methylated [4]. At the DNA level, methylation may be responsible for the regulation of gene expression [5], plant development [6] or responses to abiotic stresses [7]. Thus, alterations in DNA methylation patterns may result in either morphological [8], physiological [9] or biochemical changes [10]. They may be exhibited either among regenerants and/or their generative progenies [11, 12]. Such epimutations may arise “spontaneously” and do not appear to follow the Mendelian rules of inheritance [13, 14].

In tissue culture, reprogramming of cells (via demethylation and de novo methylation) [15] is required to force plant regeneration [16]. The mode of plant regeneration via andro- and embryogenesis is affected by the ploidy level of the source tissue [17]. The absence or duration of the callus phase may modify the reprogramming processes [18] and regeneration via tissue culture is often triggered by abiotic stresses [19]. These may alter the DNA methylation pattern [20] and activate mobile elements [21]. Finally, sequence variation may be expected [22]. Thus, studies on DNA methylation in tissue culture may generate information on epigenetic processes induced during plant regeneration and could be used as an indicator of TEs activity [23, 24].

One of the approaches to such studies is the employment of the RP-HPLC [25]. This technique delivers information on global DNA methylation in plant genomes [26]. Experiments in oil palm (*Elaeis guineensis*) demonstrated that DNA methylation of ortet (parent plant) vs. regenerants was 17.26 vs. 16.88 %, respectively [27]. The corresponding data for the in vitro derived banana (*Musa* AAA) compared to conventionally propagated plants equaled to 17.7 and 22.5 %, respectively [28]. Sianipar et al. [8] found a 2.72 % drop of global methylation between mother plants of oil palm and embryogenesis-derived progeny. In cedar (*Cedrus* sp.), a 5.6 % decrease in DNA methylation was noted among regenerants during in vitro culture about the donor plants [29]. The same trend was seen in triticale (x*Tritico secale* Wittm. ex A. Camus), where tissue culture induced a decrease in DNA methylation of the regenerants compare to donor plants [30]. The RFLP analysis based on the *Hpa*II and *Msp*I digests are in good agreement with presented data in the case of maize regenerants derived from embryos and their sexual progeny [31]. Evidently, tissue culture may induce epigenetic changes that influence not only regenerants but also their progeny.

Changes in DNA methylation are supposed to be closely related to the movement of the mobile elements [32]. On one side, it was demonstrated that a decrease in DNA methylation resulted in activation of TEs during tissue culture plant regeneration [33]. On the other hand, it has been suggested that either transposon movement is not related to tissue cultures [34], or that some transposons could be activated [35], whereas others are not, by in vitro tissue culture treatment [36]. Possibly, some transposons may be activated in response to the given stresses [37] whereas others are activated in other cases [38]. Although retrotransposon activity is considered to be one of the causes of variability induced in tissue cultures, it should be emphasized that they can also be responsible for pre-existing variation [39]. Among TEs with the activity that can be studied in tissue culture manipulations in cereals, a suitable candidate seems to be the group with the long terminal repeats (LTR) and the non-LTR retrotransposons—both of them are present in monocot and dicot angiosperms [40, 41]. One of the members of these retrotransposons, BARE-1, has homologues in different species e.g. barley, oat, wheat or rice [42, 43]. It has proven useful for detecting polymorphism in cereals [44].

To study TEs movement, one may apply techniques directed towards retrotransposon sequences e.g. inter retrotransposons amplification polymorphism (IRAP) [22], retrotransposons microsatellite amplified polymorphism (REMAP) [45], sequence specific amplified polymorphism (SSAP) [46] or methyl-sensitive transposon display (MSTD) [32] techniques. The IRAP technique was used to study sequence variation between parental plants and regenerants in three barley cultivars [44]. It was demonstrated that 29 % of cv. Golden Promise, 53 % of Tallon and 96 % of Mackay regenerants obtained via somatic embryogenesis differed from their parental forms. Similar studies with SSAP resulted in 19.66 % polymorphism between the donor plants, two callus-pools and eight regenerants developed from young inflorescence-derived calli of barley plants (*H. brevisbulatum*) [46]. In extension to IRAP and SSAP that can assess sequence changes, the MSTD based on the metAFLP approach [1, 30, 47] seems to be useful in similar studies.

Besides techniques suitable to study TEs movement in tissue culture conditions, the need of suitably derived plant material to test the putative impact of in vitro conditions for the epigenetic status of regenerated plants and their sexual progeny with the reference to TE activation and DNA methylation changes still remains. Evidently, the source of explants and donor plants, should not be affected (or such an influence should be minimized) by tissue culture itself. Possibly, this could be accomplished via using generative progeny of the DH plants [48]. However, to our best knowledge, there is no information available how many generative cycles are needed to stabilize/eliminate (if possible at all) the effects induced in tissue cultures. Nevertheless, it was shown that in triticale [30] regenerants derived via anther cultures started to reestablish their methylation status after one/two cycles. Similar data are not available in barley. However, it was shown [1] that donors derived from the progeny of DH plants were uniform both at the DNA and DNA methylation levels. Thus, to stabilize DNA methylation changes induced in regenerants during in vitro tissue cultures one should consider to use as a source of explants the tissues from the progeny of the regenerants. Obviously, control of the TEs movement is hardly possible; however, it was suggested that their movement could be controlled via generative cycle [48] giving the opportunity to study the linkage between TEs activity and DNA methylation.

The linkage between DNA methylation change due to in vitro tissue culture plant regeneration and the activation of transposons (e.g. Ty-1 Copia LTR family) is not entirely understood. It is not also clear whether the level TEs in Ds, Rs and Ps would be at the comparable level or not. The aim of the study was to verify whether different TEs families are being activated during in vitro tissue culture plant regeneration; whether their activity is transmitted to sexual progeny and whether DNA methylation is linked to TEs.

## Results

Barley regenerants obtained via andro- and embryogenesis and used in the analysis were fully self-fertile. They did not exhibit any apparent morphological changes relative to the donor plants. Similarly, no visible changes in plant morphology or growth patterns were observed for sexual progenies of the regenerants.

### RP-HPLC

RP-HPLC allowed the identification of peaks related to dC and 5mdC with retention times equal to 6.83 and 9.68 min, respectively. The average total amount of cytosine (dC + 5mdC) in the barley genome of Ds, Rs and Ps amounted to 23.38 %. The lowest mean value of the global methylation was observed in the donor plants (17.86 %), whereas the mean value in the regenerants and their progenies was 20 and 20.13 %, respectively (Table 1).

**Table 1:** DNA methylation content (global methylation) based on RP-HPLC analyses

Plant material	Global DNA methylation (%) ± SD
Ds, donor plants	17.86 ± 1.58
Rs, regenerants	20.0 ± 1.17
RE, embryogenic regenerants	20.1 ± 0.89
RA, androgenic regenerants	19.88 ± 1.43
Ps, progeny	20.13 ± 1.46
PE, progeny of embryogenic regenerants	20.17 ± 1.36
PA, progeny of androgenic regenerants	20.09 ± 1.59

*SD* standard deviation

The RP-HPLC analysis of the regenerants from any of the two regeneration approaches showed that the level of the cytosine methylation of the embryogenic (RE) and androgenic (RA) derived regenerants was 20.1 and 19.88 %, whereas DNA methylation of their progenies was 20.17 and 20.09 % for PE and PA, respectively.

ANOVA showed the increase of global DNA methylation among the regenerants relative to the donor plants (F = 36.69; *p* < 0.0001, α = 0.01), whereas DNA methylation of the generative progeny was at the same level as that for the regenerants (F = 0.28; *p* = 0.60, α = 0.05) and varied from the donors (F = 26.46; *p* < 0.0001, α = 0.01). There were no differences between the two modes of plant regeneration (androgenesis vs. somatic embryogenesis) in the global DNA methylation levels (F = 0.61; *p* = 0.44, α = 0.05). Also, the difference between progenies derived from the two types of regenerants was insignificant (F = 0.04; *p* = 0.85, α = 0.05). There were no significant alterations among individual regenerants (F = 1.73; *p* = 0.15, α = 0.05) and the progenies (F = 0.66; *p* = 0.58, α = 0.05).

### metAFLP

**Fig. 1:**
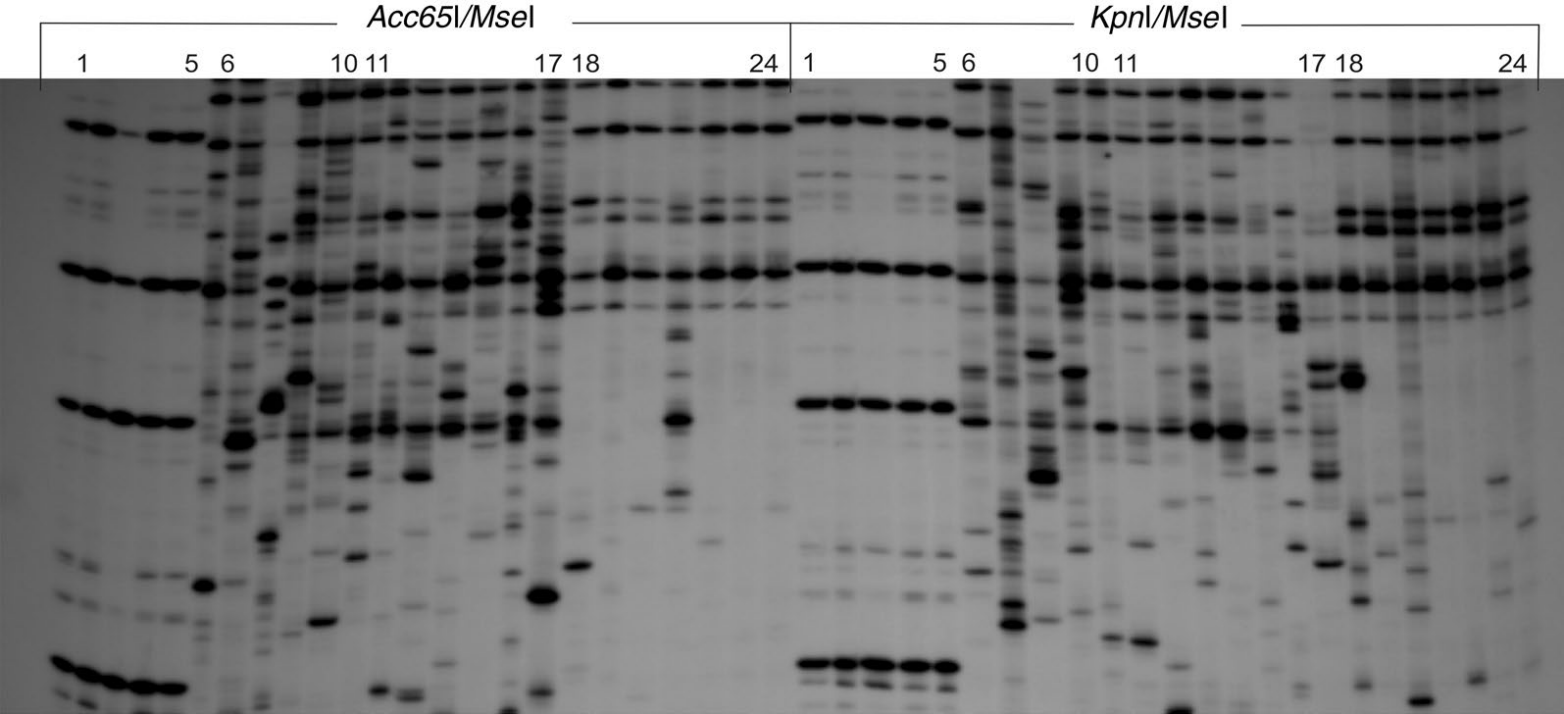
An example of the distinct SSAP profile generated with BARE1 EO377 directed and MGT selective primers. The *Acc65*I/*Mse*I (*left*) and *Kpn*I/*Mse*I (*right*) metAFLP platforms. *Lines* 6–10, 11–17 and 18–24 represent profiles of the donors, regenerants and progenies, respectively. The metAFLP pattern using CpXpG-AGC/MCGT primer pair, (*line* 1–5) representing donor plants, is included

**Fig. 2:**
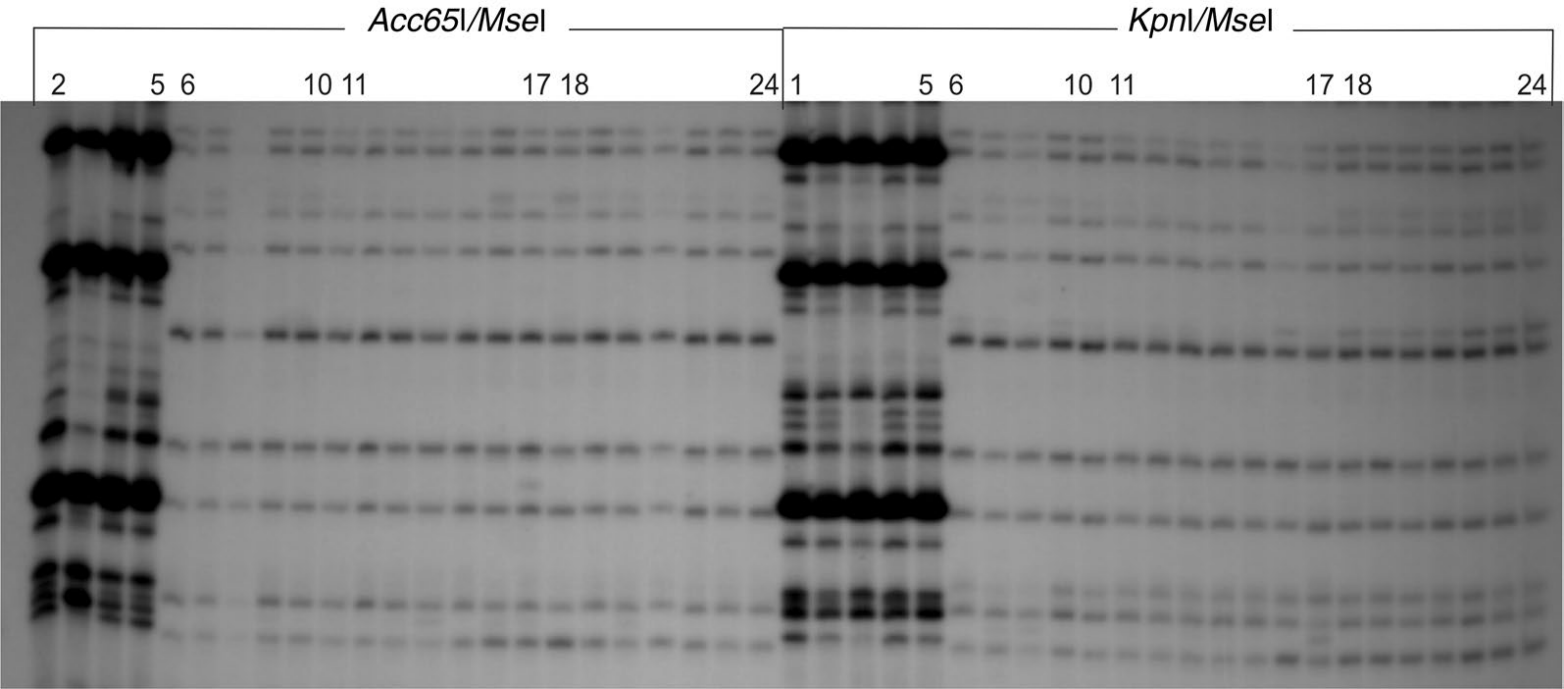
An example of the monomorphic SSAP profile generated with BARE LTR-directed and MCGT selective primers. The *Acc65*I/*Mse*I (*left*) and *Kpn*I/*Mse*I *(right*) metAFLP platforms. *Lines* 6–10, 11–17 and 18–24 represent profiles of the donors, regenerants and progenies, respectively. The metAFLP pattern using the using CpXpG-AGC/MCGT primer pair (*line* 2–5 for *Acc65*I/*Mse*I and 1–5 for *Kpn*I/*Mse*I) representing donor plants is included

The metAFLP profiling resulted in stable and highly reproducible banding patterns amplified with metAFLP selective primer pairs as illustrated in the case of donors (Figs. 1, 2).

### SSAP and MSTD markers

The primers used in SSAP and MSTD approach generated polymorphic (Fig. 1) as well as monomorphic profiles (Fig. 2). While the primers NIKITA and BARE1 EO377, resulted in highly polymorphic and hardly readable banding patterns (Fig. 1), the primers BARE LO45C, BARE LTR, SUKKULA 9900 and SUKKULA EO299 generated entirely or mostly monomorphic profiles detected for Ds, Rs and Ps samples (Fig. 2).

The SSAP and MSTD approach using finally 12 selective primer pairs amplified 513 markers with 210 and 293 being polymorphic, respectively. There were 41, 39 and ten markers amplified via the SSAP method and shared exclusively among Ds, Rs and Ps, respectively. In the MSTD, these amounts were 75, 24 and 9, respectively. The SSAP markers were less polymorphic than the MSTD ones (Table 2). Moreover, markers in the progeny were less polymorphic than those in regenerants and donors. Shannon’s information indices followed the very similar pattern of changes in both approaches with the highest values for the donors and the lowest for the progenies.

**Table 2:** The arrangement of the SSAP and MSTD data

Method	Plant materials					
	Donors		Regenerants		Progeny	
	*P* %	*I*	*P* %	*I*	*P* %	*I*
SSAP	28.65	0.132	24.17	0.094	11.50	0.046
MSTD	46.0	0.215	34.11	0.141	21.83	0.081

*P* % is the percentage of polymorphic loci, *I* Shannon’s information index

Analysis of Molecular Variance demonstrated that the difference between Ds, Rs and Ps evaluated based on both approaches were significant (Table 3). The difference between Ds, Rs and Ps was also assessed for SSAP profiles (Φ_*PT*_ = 0.289, *p* = 0.001) and MSTD data (Φ_*PT*_ = 0.199, *p* = 0.001). Comparison of the SSAP data pointed at R–P as the one with the highest value of the variance (Table 3). The same comparison (R–P) for MSTD did not reach as high value as for SSAP. The highest Φ_*PT*_ value contrasted D-P for MSTD markers (Table 3). Φ_*PT*_ values demonstrated that explained variance between Rs and Ps was greater than that between Ds and Ps and between Ds and Rs based on SSAP markers. The explained variance was the highest between Ds and Ps and the lowest between Ds and Rs in the case of the MSTD markers.

**Table 3:** The arrangement of the molecular variance evaluated for the comparisons of D–R, D–P and R–P (donor, regenerant, progeny) based on SSAP and MSTD data

Method	*Φ*_*PT*_ value		
	Donors–regenerant	Donors–progeny	Regenerants–progeny
SSAP	0.155 (*p* = 0.002)	0.283 (*p* = 0.003)	0.408 (*p* = 0.001)
MSTD	0.107 (*p* = 0.001)	0.269 (*p* = 0.002)	0.228 (*p* = 0.001)

*Φ*_*PT*_ values evaluated among donors, regenerants and progeny based on the SSAP and MSTD profiles

**Fig. 3:**
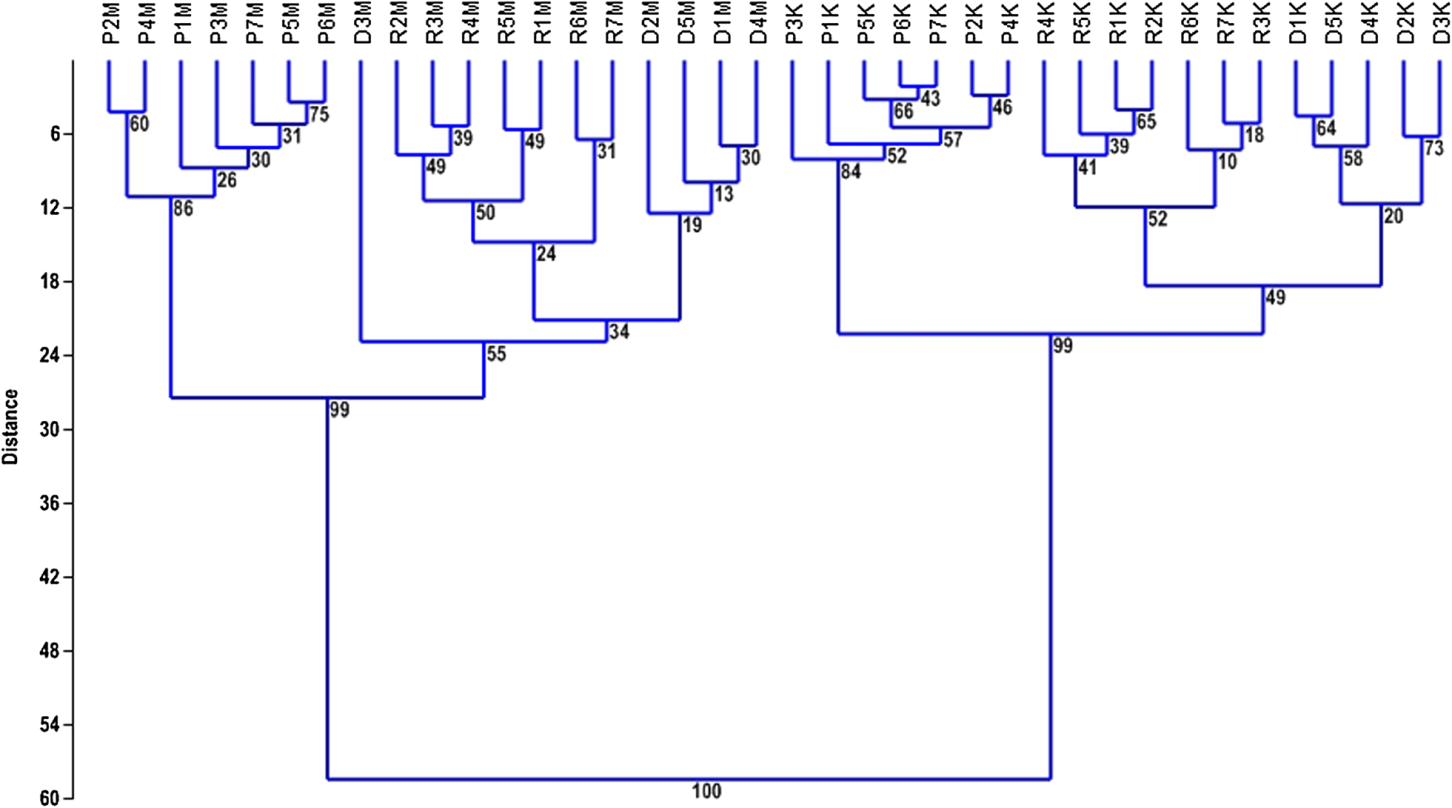
Clustering of Ds, Rs, and Ps based on SSAP and MSTD markers using Ward’s method with bootstrap values at the nodes. D, R, and P reflect donors, regenerants and their sexual progeny; “K” states for the SSAP whereas “M” for the MSTD results

Ward’s method of clustering based on SSAP and MSTD markers divided the data into two independent parts. The first one reflected the results assessed via the SSAP and the second via MSTD approach. The SSAP method classified Ds, Rs, and Ps into three distinct groups (Fig. 3). The presented analysis pointed at donors and regenerants exhibiting the highest diversity compared to the progeny. However, a difference in variation between Rs and Ds was either negligible or slightly increased in Rs. The MSTD method resulted in one cluster encompassing of progeny and the other represented by donors and regenerants. The difference among regenerants was greater than among donors. Independently of the marker system, the variation level among sexual progeny was lower than that among donor plants and their regenerants. Moreover, the SSAP revealed smaller variation level among Ds, Rs, and Ps than the MSTD.

## Discussion

Visual inspection of Rs and Ps failed to identify any morphological differences among analyzed plants and all of them were in donor plant type suggesting the lack of the tissue culture induced and somaclonal variation. However, this result is in contrast to the data evaluated based on RP-HPLC approach indicating the increase in DNA methylation of the regenerants conserved in the progeny comparing to the donors. A similar change in the global DNA methylation related to in vitro regeneration, detected by the RP-HPLC method, was observed in oil palm (*Elaeis guineensis*) regenerants [49] as well as in *Gentiana pannonica* [50]. On the other hand, a decrease was found in triticale (x*Tritico secale* Wittm. ex A. Camus) [30]. Thus, at least two alternative pathways of DNA methylation under tissue culture condition appear possible. In the first scenario, tissue culture induces an increase of DNA methylation of regenerants whereas in the second one demethylation is observed. It could be speculated that there might be some differences in demethylation and de novo methylation of species under tissue culture conditions. Alternatively, some differences could be related to the ploidy levels. It appears that at least some diploids (e.g. barley) would tend to increase their DNA methylation levels while polyploids (e.g. triticale) decrease their levels under tissue culture conditions. However, the difference between barley and triticale may also reflect the general instability of triticale [51]. The instability may be related to delayed replication of rye chromosomes [52]. However, available data [53] seems to indicate that the pathway is more species rather than the ploidy level specific. Such a notion appears to be true as the tested species at the same ploidy level (barley, oil palm) may either be affected by prevailing de novo methylation or demethylation of genomic DNA of the regenerated plants [29, 46, 49]. Interestingly, as the RP-HPLC delivers averaged information on DNA methylation change it may not reflect subtle alterations identified by marker-based approaches. Thus, in barley, the MSAP method [46] showed a 3 % drop in global DNA methylation of the restriction sites observed in Rs compared to Ds whereas metAFLP [1] demonstrated that both site DNA de novo methylation and demethylation changes nearly equally affected regenerants in comparison to donors. On the other hand, the RP-HPLC and metAFLP results assessed in *Gentiana pannonica* clearly demonstrated the increase in genomic methylation of the regenerants [50]. The differences between molecular approaches may suggest varying distribution of the sites or their presence in genome regions distinctly affected by DNA methylation changes because the MSAP approach is based on *Hpa*II and *Msp*I whereas metAFLP on *Acc65*I and *Msp*I isoschizomers. Such a notion seems to be supported by the uneven distribution of AFLPs on genetic maps of wheat [54] and rye [55] which could also be the case in barley. Thus, molecular marker approaches could be valuable tools to study fluctuations in site DNA methylation pattern changes or those bind to specific genomic elements (i.e. transposons, genes).

It has been suggested that demethylation due to abiotic stresses may be responsible for the activation of mobile elements [56]. As metAFLP proved to be useful for the analysis of methylation changes, a slight modification that could utilize selective primers towards varied classes of TEs could be a method of choice. The most common retrotransposons shared among cereals are those classified as LTR [57, 58] and solo LTR families [59]. The former are represented by BARE-1, whereas the later by NIKITA and SUKKULA [59]. It was expected that utilization of the selective primers directed towards BARE-1, NIKITA and SUKKULA TE sequences in the SSAP and MSTD method would help in linking methylation changes among Ds, Rs and Ps and alterations in DNA sequences. Hierarchical clustering demonstrated that whereas Ds, Rs and Ps formed separate groups based on the SSAP approach, the level of genetic variation in Rs was slightly higher or nearly identical to that in Ds whereas in Ps was lower than in former cases. Similarly, the MSTD analysis confirmed more moderate variation among Ps than that among Rs and Ds individuals that formed the separate cluster. However, Rs exhibited higher variation than Ds. The differences among Rs identified by the MSTD are even greater than Rs produced by SSAP approach. The contrast between the three groups of plants is also evidenced by AMOVA and confirms that TEs activity exhibited among Rs could be related to DNA methylation changes induced in tissue cultures. Moreover, higher variation level among Rs assessed by the MSTD method than by the SSAP one is in agreement with our results in barley demonstrating that in vitro tissue cultures induce increased site DNA methylation compared to sequence changes [1] which could be linked to TEs activation in tissue cultures. The observed phenomenon is congruent with the data presented by the others [60, 61].

Our former study [1], as well as current analysis of donor plants based on the metAFLP approach, demonstrated that Ds were highly uniform at the (epi)genetic level. Moreover, independently of whether the SSAP or MSTD approach was applied the variation among Ds is evident. The level of DNA sequence polymorphisms among Ds (evaluated by the SSAP) was nearly identical to that observed among regenerants of the randomly chosen donor (as indicated e.g. by cluster analysis). It should be stressed that donors were the progeny of the tissue culture regenerated plants. Obviously, the donors could be affected by TEs movement (illustrated by SSAP and MSTD approaches) influencing our estimation of TE related variation. Alternatively, the difference between donors could be explained by the so-called pre-existing variation [39] that resulted in TE-related variation not revealed by the metAFLP approach. It is being suggested, however, that generative cycle should stabilize putative TE activity [48]. Thus, individual donor plants should be treated as uniform materials.

As individual donor plants were assumed to be a homozygous generative progeny of DH regenerants (see “Methods” section) they are adequate material for studying TEs movement due to in vitro tissue plant regeneration. Thus, most—if not all—of the TE-related variation observed in Rs (and transmitted to Ps) comparing to the given D plant was the result of the tissue culture plant regeneration. Alternatively, the polymorphic TE-related profiles revealed for Rs could be interpreted regarding background activity of some mobile elements, which to some extent may be responsible for the pre-existing variation not related to the in vitro tissue culture plant regeneration. If so, then, at least, some retrotransposons may generate variation that is not related to in vitro tissue culture.

Interestingly, some primers directed towards BARE-1and NIKITA TEs failed to amplify whereas those towards i.e. SUKKULA and the other BARE-1 amplified hardly polymorphic profiles. Thus, we tend to think that some TEs could be active whereas the others not under tissue culture plant regeneration. Such results could be explained by the different activity of various mobile elements [62] or even altered action of the same TE in distinct species due to e.g. stresses like tissue culture [35, 36]. Our data is in agreement with previous reports, indicating that distinct TEs could be activated due to in vitro tissue culture plant regeneration [35, 63].

Although we cannot entirely exclude that a decrease in genetic variation in Ps compared to Ds and Rs is due to the progeny that originated from a single regenerant, we suggest that TEs movement detected among Ps is reduced regarding sexual reproduction. This notion is supported by polymorphisms revealed via the MSTD and SSAP that decreased in Ps in parallel to a nearly identical level of DNA methylation of Ps and Rs as assessed by RP-HPLC approach. Thus, our results favor the hypothesis of TE stabilization due to sexual reproduction [48] indicating that reproductive cycle of in vitro regenerated DH plants may somewhat limit the level of variation related to retrotransposon activity (making such materials suitable for studies on TE related changes originating due to tissue culture plant regeneration methods). However, independent studies are needed to verify how many reproductive cycles are needed to eliminate/minimize the TEs activation due to tissue culture manipulations and whether this is dependent on species and mode of reproduction.

According to a common notion, plant regeneration via androgenesis should be less error prone than via embryogenesis due to the lack or hardly observed callus phase [64, 65], which is responsible for the release of cellular control over mutagenesis generated i.e. by the motion of transposons agents possibly induced as the result of genomic DNA demethylation [66]. The RP-HPLC approach, however, failed to assess differences in DNA methylation due to the mode of the in vitro tissue culture plant regeneration which is congruent with our previous studies in barley [1] based on the metAFLP approach, and those in triticale [47]. Thus, the presence of putative callus phase during embryogenesis is not the source of the tissue culture-induced variation. Alternatively, other factors (i.e. haploid and the diploid fabrics) diminished the level of variation in the case of embryogenesis derived regenerants [1]. Finally, we cannot exclude that, general metAFLP characteristics failed to detect subtle changes that were assessed based on the advanced ones evaluated in the case of triticale [47] demonstrating that the mode of plant regeneration could influence tissue culture-induced variation.

## Conclusion

Stressful conditions during plant regeneration via in vitro tissue cultures affect regenerants and their sexual progeny leading to an increase in global DNA methylation of Rs and Ds compared to Ds in barley. The increased methylation level revealed among regenerants remains unchanged in the Ps as indicated via RP-HPLC data. It is usually assumed that such a change in methylation is related to stabilization of the TEs activity. Our marker based experiments showed that TEs seem to be activated via in vitro tissue cultures and that independently of the increased methylation their activity in Rs is greater than in Ps. Thus, the increased methylation level may not correspond to the stabilization of TEs movement at least at the level of regenerants. We have also shown the presence of TEs variation among Ds that were genetically and epigenetically uniform as indicated by the metAFLP approach. TEs variation assessed among Ds may suggest that at least some mobile elements may be active, and they may mask variation related to tissue cultures. It should be stressed however, that we cannot exclude that the donor plants being the progeny of DH regenerants failed to stabilize TEs activity most probably induced during tissue culture manipulations. Thus, tissue cultures may activate some TEs whereas the others remain intact, or their level of movement is changed. Moreover, sexual reproduction may be responsible for the stabilization of TEs (possibly due to stabilized methylation of the genome). Nevertheless, their movement is not eliminated.

## Methods

### Plant materials for RP-HPLC

**Fig. 4:**
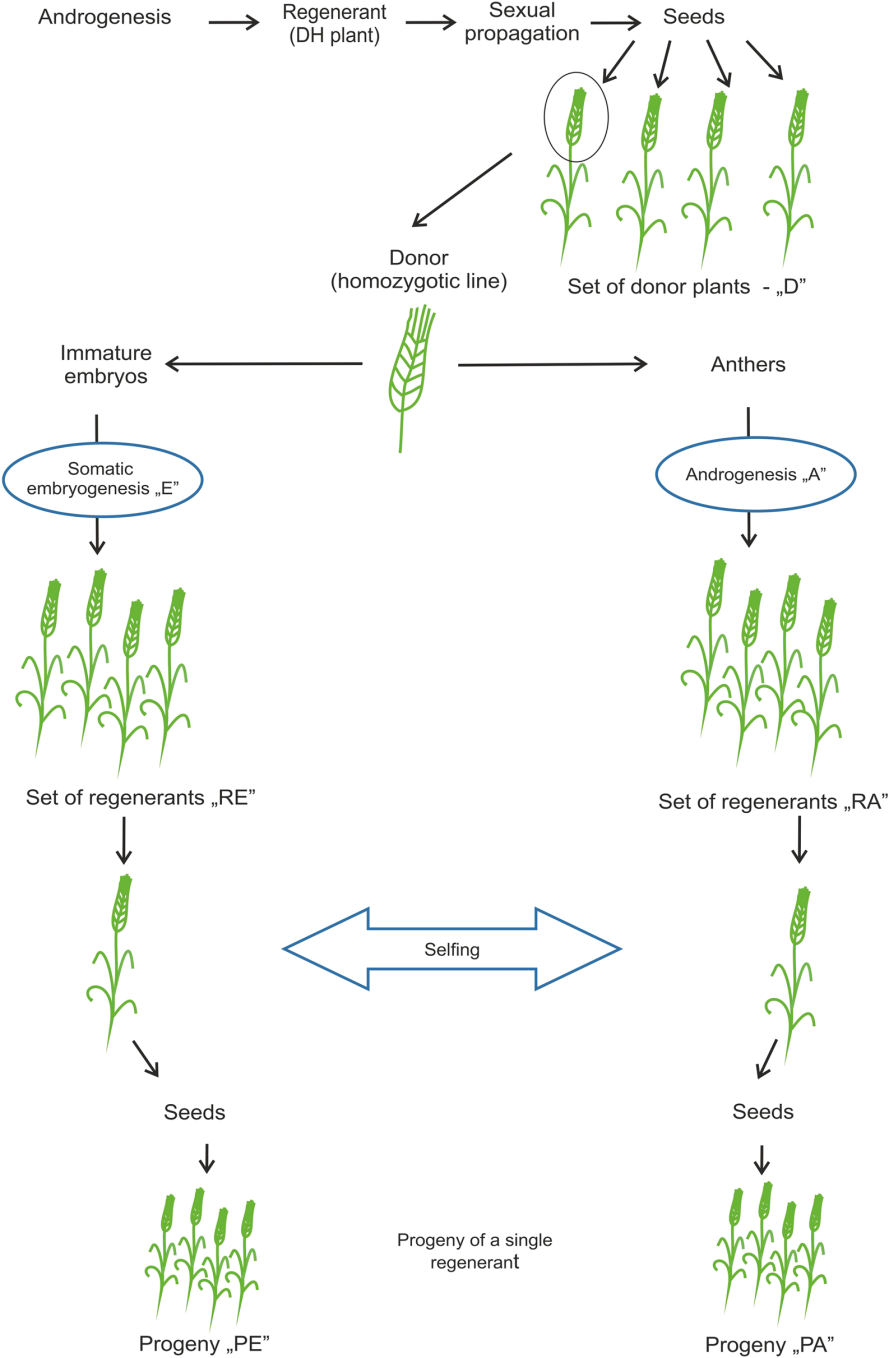
A schematic representation of the plant materials. *D* stands for donor plants, *R* for regenerants and *P* for progeny. *RE* and *RA* refer to regenerants obtained via somatic embryogenesis and androgenesis, respectively, whereas *PE* and *PA* are the progenies of regenerants obtained via somatic embryogenesis and androgenesis, respectively

The starting materials were full-sib progenies (Ds, donor plants; 22 plants) of individual doubled haploid (DH) barley (*Hordeum vulgare* L.; cv. Scarlett) plants. The DH plants originated via androgenesis in isolated microspore culture [67]. Ds served as a source of explants to obtain regenerants (Rs) either by androgenesis in anther cultures (labeled RA, 35 plants) or by somatic embryogenesis using immature zygotic embryo cultures (labeled RE, 37 plants). Randomly chosen regenerants (four from androgenesis and four from embryogenesis) were self-pollinated to obtain sexual progenies (labeled Ps, PE, 24 plants, PA, 22 plants) (Fig. 4).

### Androgenesis

Tillers of the donor plants were collected at late microspore stage and kept for 3 weeks in water in darkness at 4 °C. Spikes were surface sterilized, anthers removed from the spikes and plated on solidified medium N6 [68] supplemented with vitamins [69], with 2 mg l^−1^ 2,4-D, 0.5 mg l^−1^ kinetin and 80 g l^−1^ maltose. They were cultured in Petri dishes in the dark at 26 °C for 4–6 weeks. Androgenic structures (calli, embryos) were transferred to the regeneration medium 190-2 [70] supplemented with 0.5 mg l^−1^ NAA and 0.5 mg l^−1^ kinetin [71]. Cultures were kept under 16/8 h (day/night photoperiod) for 2–4 weeks. Green plantlets were transferred to half-strength MS [72] medium without growth hormones for rooting. Plants were potted, adapted to soil conditions and grown in the greenhouse to maturity under standard conditions.

### Somatic embryogenesis

Embryos were excised from immature sterilized caryopses from donor plants (harvested 12–16 days after pollination), plated on MS medium supplemented with 2 mg l ^−1^ 2,4-D [73] with the scutellum sidefacing up. The plates were incubated under a 16/8 h (day/night) photoperiod at 26 °C for 3–4 weeks. Embryogenic calli were transferred to regeneration media (the same as for androgenesis) and subcultured every 2 weeks. Rooted plants were transferred to the greenhouse and grown to maturity under standard conditions.

### Progeny of regenerants

Four regenerants from androgenesis and four from embryogenesis were self-pollinated. These regenerants were derived from four different donor plants (two regenerants each obtained from one donor plant). Separated seed samples of these eight regenerants were used to derive the first generation progenies of the regenerants (Ps) (Fig. 4).

### Plant materials for SSAP and MSTD

Five donor plants (the progeny of DH regenerants), seven regenerants derived via somatic embryogenesis from one of the donor plants and seven progenies obtained from one regenerant was the plant material used in SSAP and MSTD approach. All plants were chosen from those prepared for RP-HPLC analysis.

### Genomic DNA extraction

Total genomic DNA was isolated from 100 mg of 7-day-old seedling leaves, using the DNasy Mini Prepkit (Qiagen GmbH, Hilden, Germany). The quantity of DNA was evaluated spectrophotometrically at λ = 260 nm. DNA integrity and purity was verified electrophoretically on 1.2 % agarose gel in 1× TBE, stained with ethidium bromide of final concentration 0.5 μg ml^−1^. Separation was performed at 160 V for 30 min.

### DNA preparation to RP-HPLC

DNA samples (4 μg each) were dried, dissolved in 100 μl of deionised water, denaturated (100 °C for 2 min) and left on ice for 5 min. The mixture was gently stirred after adding 5 μl of 10 mM ZnSO_4_and 10 μl of 1.0 U ml^−1^ nuclease P1 in 30 mM NaOAc (pH 5.4) and then incubated at 37 °C for 17 h. After incubation, 10 μl of 0.5 M Tris (pH 8.3) and 10 μl of 10.0 U ml^−1^ alkaline phosphatase in 2.5 M (NH_4_)_2_SO_4_ were added, and samples were again gently stirred and incubated at 37 °C for 2 h. Samples were centrifuged for 5 min at 12 × 10^3^ rpm.

### RP-HPLC c12

RP-HPLC analysis was performed using the Waters 625 LC System (encompassing: Waters 625 Pump, Waters 600 Controller, Waters 717plus Autosampler, Waters Degasser and Waters 996 PDA detector) Synergy Max-RP C12 (250 × 4.6 mm, 4u, Phenomenex) column, combined with Synergy Max-RP C12 pre-column according to an adapted procedure [74, 75]. Separation of nucleosides was conducted in the presence of ‘A’ buffer (0.5 % v/v methanol in 10 mM KH_2_PO_4_, pH 3.7) and ‘B’ buffer (10 % v/v methanol in 10 mM KH_2_PO_4_, pH 3.7). The pH of the buffers was adjusted with phosphoric acid. The linear gradient used for separation consisted of 100 % ‘A’ buffer to 100 % of ‘B’ buffer for 10 min, next 100 % of ‘B’ buffer for 10–25 min and then, at the end of 25 min program 100 % ‘A’ buffer was pumped for 5 min. Flow rate was 1 ml per min and column temperature was set at 30 °C. UV-detection was used at the wavelength of 280 nm. The external standard consisted of major DNA (0.5–50 µM) and RNA nucleosides (1.5–150 µM) and 5-methyl-2′-deoxycytidine (5mdC) dissolved in deionized water. Peaks corresponding to 2′-deoxycytidine (dC) and 5mdC had retention time equal to 6.5 and 9.3 min, respectively. The contribution of 5mdC was calculated based on Millennium 32 v. 4.0 software (Waters Corporation, Milford, Massachusetts, USA).

### Assessment of nucleosides

Quantification of nucleosides was based on the automatically integrated surface areas (μV s^−1^) of the chromatograph peaks. The amount of cytidine (dC) and 5-methyl-2′-deoxycytidine (5mdC) (also sum of dC and 5mdC) in relation to all nucleosides was assessed using the formula: ${\text{dC}} = {{\text{dC}} \mathord{\left/ {\vphantom {{\text{dC}} {\left( {{\text{dC}} + 5{\text{mdc}} + {\text{dG}} + {\text{dT}} + {\text{dA}}} \right)}}} \right. \kern-0pt} {\left( {{\text{dC}} + 5{\text{mdc}} + {\text{dG}} + {\text{dT}} + {\text{dA}}} \right)}} \times 100$ and $5{\text{mdc}} = {{5{\text{mdc}}} \mathord{\left/ {\vphantom {{5{\text{mdc}}} {\left( {{\text{dC}} + 5{\text{mdc}} + {\text{dG}} + {\text{dT}} + {\text{dA}}} \right)}}} \right. \kern-0pt} {\left( {{\text{dC}} + 5{\text{mdc}} + {\text{dG}} + {\text{dT}} + {\text{dA}}} \right)}} \times 100.$ The amount of global DNA methylation was calculated as the concentration of 5-methyl-2′-deoxycytidine (5mdC) in relation to the whole amount of cytidine according to the formula: ${{5{\text{mdc}}} \mathord{\left/ {\vphantom {{5{\text{mdc}}} {\left( {5{\text{mdc}} + {\text{dC}}} \right)}}} \right. \kern-0pt} {\left( {5{\text{mdc}} + {\text{dC}}} \right)}} \times 100$. Mean values and standard deviation of the amount of global DNA methylation were evaluated for D, R, P, RA, RE, PA and PE.

### MetAFLP approach

The metAFLP procedure followed that described elsewhere [1]. The arrangement of selective primer combinations is given in Table 4.

**Table 4:** The metAFLP, SSAP and MSTD primer sequences

Selective primer	Selective primer combinations 5′→3′
	*Mse*I directed primer sequences
MCAA	GAT GAG TCC TGA GTA ACA A
MCAG	GAT GAG TCC TGA GTA ACA G
MCCA	GAT GAG TCC TGA GTA ACC A
MCCG	GAT GAG TCC TGA GTA ACC G
MCGT	GAT GAG TCC TGA GTA ACG T
MCTC	GAT GAG TCC TGA GTA ACT C
	metAFLP directed selective primer sequences
CpXpG-AGC	CAT GCG TAC AGT ACC AGC
CpG-GCA	CA TGC GTA CAG TAC CGC A
	TE directed selective primer sequences
BARE1 E0377	TGT TGG AAT TAT GCC CTA GAG G
BARE LO45C	TGT TTC CCA TGC GAC GTT CC
BARE LTR	CTA GGG CAT AAT TCC AAC A
BARE1 E1814	TTG CCA TGC GAC GTT CCC CAA C
BARE1 92460	CTG GCT AGC CAA CTA GAG GCT TGC
BARE1 81078	ATC ATT GCC TCT AGG GCA TAA TTC C
SUKKULA 9900	GAT AGG GTC GCA TCT TGG GCG TGA C
SUKKULA E0229	ACG TCG GCA TCG GGC TGT CAC
SUKKULA E0228	GGA ACG TCG GCA TCG GGC TG
NIKITA E2611	TGG GAT CAC TTG ATC CCT CTC G
NIKITA	AAG AAG TGC CTA TGG ACA AAT CC

### Sequence-specific amplification polymorphism (SSAP) based on met AFLP platforms

The SSAP approach was based on the metAFLP technique [1]. The DNA samples were digested with the *Kpn*I and *Mse*I endonucleases, following adaptor ligation, pre-selective and selective amplification steps. For the selective amplification step oligonucleotides directed toward BARE-1, NIKITA and SUKKULA sequences and *Mse*I adaptor sequences (Table 4) were used. The selective amplification was followed by electrophoresis on 7 % PAGE and exposure to X-ray film.

### Methyl-sensitive transposon display (MSTD) based on metAFLP platforms

The Methyl-sensitive transposon display (MSTD) was based on the metAFLP approach [1]. The markers related to DNA methylation were extracted following the procedure described by Chwedorzewska & Bednarek [76]. Briefly, molecular profiles based on the *Acc65*I/*Mse*I (A) and *Kpn*I/*Mse*I (K) platforms were juxtaposed and scored in a ‘0–1’ binary matrix with ‘1’ standing for the presence and ‘0’ for the absence of the marker. As the *Acc65*I/*Mse*I platform is capable of identifying (epi)mutations while *Kpn*I/*Mse*I one only sequence changes, this information was used to extract epimarkers. Markers that were present in the first and missed in the second (or vice versa) metAFLP platform were related to DNA methylation *Acc65*I/*MseI*-*Kpn*I/*Mse*I (A–K). Thus, the data from both platforms were used to evaluate the “DNA methylation associated markers” also described as “epimarkers”. Instead of the *Mse*I selective primers applied in metAFLP method, those directed towards BARE-1, NIKITA and SUKKULA elements combined with primers directed to methylation—CpGand CpXpG (Table 4) were used to reflect TEs activity. The other steps were performed as for SSAP.

### Statistics

Analysis of variance (ANOVA) was applied for the RP-HPLC results using SAS software version 9.1 [77].

GenAlEx6.501 (Excel add-in software) [78] was used to estimate: the number of bands, the number of unique (individual) bands, a percentage of polymorphic loci (*P* %) generated by metAFLP platforms. Shannon’s diversity index (*I*) was applied to characterize marker informativeness. PAST software [79] was used for cluster analysis (Ward’s method) with 1000 bootstrap replicates to estimate the robustness of the branches. Analysis of Molecular Variance—AMOVA (Φ_*PT*_ index value) in GenAlEx6.501 was applied to SSAP and MSTD data. Reliability of the results was performed using 999 random permutations of the raw data.

## Abbreviations

2,4-D: 2,4-dichlorophenoxyacetic acid
NAA: 1-naphthaleneacetic acid
5mdC: 5-methyl-2’-deoxycytidine
MS: Murashige and Skoog basal salt mixture
MSAP: methylation sensitive amplified polymorphism
RFLP: restriction fragments length polymorphism
RP-HPLC: reverse phase-high performance liquid chromatography
SSAP: sequence-specific amplification polymorphism
MSTD: methyl-sensitive transposon display
TE: transposable element
